# Advances in the pathogenesis of rosacea

**DOI:** 10.3389/fimmu.2025.1705588

**Published:** 2026-01-21

**Authors:** Hui Wang, Cheng Zhou

**Affiliations:** Department of Dermatology, Peking University People’s Hospital, Beijing, China

**Keywords:** genetics, immunology, microbiome, neurovascular, pathogenesis, rosacea

## Abstract

Rosacea is a chronic inflammatory cutaneous disorder predominantly affecting the centrofacial region, whose pathogenesis is complex and not yet fully understood. In this review we summarized the latest significant advances in the pathogenesis of rosacea in recent years. In genomic studies, the application of bioinformatics techniques such as whole-genome sequencing has identified novel susceptibility genes and linked multiple pathogenic mechanisms. Neurovascular dysfunction resulting from abnormal neuropeptides expression and dysregulated amino acid metabolism constitutes an important pathogenic factor in rosacea. The TLR2/LL-37/mTORC1 signaling axis, as a core regulatory pathway in innate immunity has been elucidated in detail. In addition, the dysbiosis of skin and gut microbiota, together with the impairment of skin barrier function, is also closely associated with the onset and progression of this disease. The deeper understanding of the pathogenesis of rosacea will benefit the development of new drugs and promote individualized diagnosis and treatment.

## 1 Introduction

Rosacea is a chronic inflammatory dermatosis characterized by centrofacial erythema, telangiectasia, papulopustular lesions, and phymatous changes (1, 2). Global prevalence estimates range from 1% to 20% based on population studies, with higher rates typically observed in fair-skinned populations of Northern European descent. The overall prevalence of rosacea is 5.1% (3, 4). Rosacea predominantly affects women aged 20–50 years. Due to its visible impact on facial appearance, patients frequently experience embarrassment, anxiety, and diminished self-esteem, which may progress to clinical depression. These might lead to social and occupational impairment, ultimately imposing substantial negative effects on quality of life (5, 6).

Rosacea classification has transitioned from a fixed subtype-based system to a flexible phenotype-driven approach. The 2019 Global Rosacea Consensus (ROSCO 2019) emphasizes diagnosis based on observable clinical features rather than predefined subtypes. Major phenotypic traits include persistent centrofacial erythema, papules/pustules, and telangiectasia. Minor features including stinging, burning, dryness, and edema. Ocular rosacea features include lid margin telangiectasia, blepharitis, keratitis, conjunctivitis, and anterior uveitis. This shift supports individualized management and improved patient outcomes (7).

**Figure 1:**
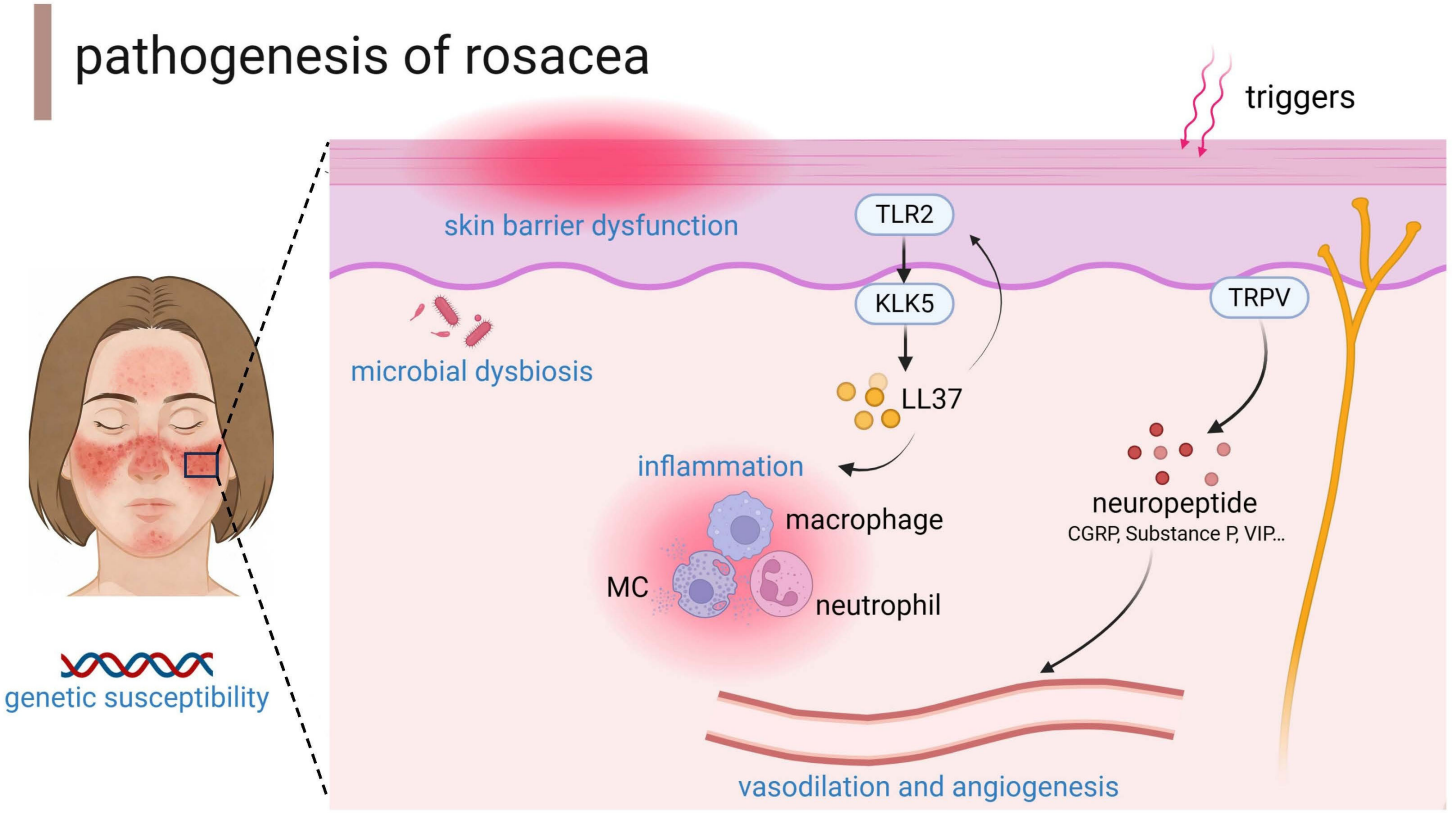
The main pathogenesis of rosacea. TLR2, toll-like receptor 2; KLK-5, kallikrein 5; MC, mast cell; TRP-, transient receptor potential channel; VIP, vasoactive intestinal polypeptide; CGRP, calcitonin gene related peptide. The figure was created with BioRender.com.

The pathophysiology of rosacea remains incompletely understood with multifactorial origins; however, ongoing research continues to delineate its underlying mechanisms. Current evidence confirms that genetic predisposition, immune dysregulation, neurovascular dysfunction, microbial factors, and environmental triggers constitute key pathogenic determinants (8, 9) (**Figure 1**). Advancing the comprehension of rosacea mechanisms is fundamental for directing rational therapeutic development. So, in this review we have comprehensively summarized the latest research progress on the pathogenesis of rosacea, providing a foundation for the development of new drugs and precise treatment.

## 2 Genetic factors

Epidemiological studies reveal significant heterogeneity in rosacea prevalence globally (3). Twin cohort analysis demonstrated significantly higher concordance in rosacea severity among monozygotic versus dizygotic twins, confirming substantial genetic contributions to disease development. Approximately 46% of the variation in rosacea severity was attributed to genetic factors. These findings indicate that genetic susceptibility is a key component in the pathogenesis of rosacea (10). Genome-wide association studies (GWAS) by Raber et al. identified significant associations between rosacea and specific human leukocyte antigen (HLA) alleles (HLA-DRB1, HLA-DQB1, HLA-DQA1, rs763035) in European populations. This finding has been corroborated by subsequent discoveries of novel rosacea-predisposing HLA variants in Chinese cohorts (11, 12). Signal transducer and activator of transcription (STAT1) activation drives production of proinflammatory cytokines - including IFN-α, IFN-γ, and IL-6 - that promote chronic facial inflammation central to rosacea pathogenesis (13, 14). Notably, Gain-of-function (GOF) mutations in STAT1 are implicated in early-onset familial rosacea (15). Another study used whole-skin RNA-seq to compare transcriptomic signatures among healthy facial skin, rosacea lesions, and patient-matched normal skin. Integrated gene ontology, pathway, and transcription factor enrichment analyses established STAT1 as a central regulator of epithelial-immune crosstalk in lesional tissue. Immunohistochemistry confirmed STAT1/IRF1 upregulation across all major rosacea subtypes, demonstrating its fundamental pathogenic role (16). Glutathione S-transferases (GSTs) play critical roles in cellular defense against electrophilic compounds and oxidative stress. Evidence suggests rosacea pathogenesis involves both increased reactive oxygen species (ROS) activity and impaired antioxidant capacity. Genotypes with concurrent GSTM1 and GSTT1 null polymorphisms confer elevated disease risk (17, 18). Vitamin D status also correlates with rosacea pathogenesis. In a cohort study, Akdogan N et al. demonstrated that vitamin D receptor (VDR) gene polymorphisms (ApaI and Cdx2) can increase rosacea susceptibility, whereas the TaqI polymorphism conversely reduces disease risk (19, 20). Recently, a Whole genome sequencing (WGS) studies have further implicated LRRC4, SH3PXD2A, and SLC26A8 gene mutations in rosacea susceptibility. These genes encode proteins integral to synaptic processes and cellular adhesion. *In vitro* functional analyses confirm that disease-associated variants in these loci induce neuropeptide-mediated vasoactive responses in human neuronal cells (21). These findings hold substantial clinical implications for personalized medicine. Genotype screening enables identification of high-risk individuals and facilitates development of immune-genotype-guided therapeutic strategies.

## 3 Neurovascular dysregulation

Neurovascular dysfunction plays a central role in the multifactorial pathogenesis of rosacea and highly associated with persistent erythema or flushing (22). This dysregulation centers on transient receptor potential (TRP) channels, which function as non-selective Ca²^+^-permeable channels with dual sensory and signaling capabilities. TRP channels are expressed across multiple cell types implicated in rosacea, including sensory neurons, MCs, endothelial cells, and keratinocytes (23). These channels respond to common rosacea triggers: for instance, activation of transient receptor potential vanilloid 1 (TRPV1) by heat, emotional stress, or capsaicin induces characteristic pain and burning sensations (24). TRP activation initiates vasodilatory pathways; TRPV4 stimulation promotes the release of nitric oxide (NO), prostaglandins (PGs), and endothelium-derived hyperpolarizing factor, exacerbating erythema and inflammation (24, 25). Leading to the release of neuropeptides such as the pituitary adenylate cyclase-activated polypeptide (PACAP), substance P, and the calcitonin gene-related peptide (CGRP) (25). These neurotransmitters can bind to receptors on immune cells, such as mast cells (MCs), macrophages, and neutrophils, thereby inducing the production of pro-inflammatory mediators (26). Deng, Z et al. integrated genomic and functional analyses have elucidated the link between rosacea and neurogenic inflammation. In a mouse model recapitulating a recurrent Lrrc4 mutation found in human patients, the animals exhibited rosacea-like cutaneous inflammation associated with excessive vasoactive intestinal peptide (VIP) release from peripheral neurons. These findings further support the important role of neurogenic inflammation in rosacea pathogenesis (21).Additionally, Liu et al. linked amino acid metabolism to neurovascular reactivity in rosacea. They identified dysregulated amino acid metabolism, particularly elevated glutamate and aspartate levels, as a key metabolic feature in rosacea patients, which correlated positively with disease severity. These amino acids promote neurovascular reactivity by stimulating the release of vasodilatory neuropeptides from neurons and keratinocytes, and by inducing NO production in endothelial cells and keratinocytes. Furthermore, doxycycline may alleviate rosacea symptoms by targeting this dysregulated amino acid metabolism, suggesting a potential therapeutic strategy (27).

## 4 Immune dysregulation

### 4.1 Innate immune dysregulation

#### 4.1.1 LTR2/LL37 pathway

Toll-like receptor 2 (TLR2), a pattern recognition receptor (PRR), activates the innate immune system by recognizing pathogen-associated molecular patterns (PAMPs) and damage-associated molecular patterns (DAMPs) (28, 29). TLR2 expressed on the plasma membrane of diverse cutaneous cell types, including keratinocytes and fibroblasts. TLRs are also expressed in immune system cells such as MCs, phagocytes, and dendritic cells (30). In rosacea lesions TLR2 is overexpressed, which promotes the production and release of the serine protease kallikrein-related peptidase 5 (KLK5) and inflammatory mediators such as the cathelicidin (LL-37), interleukin-8 (IL-8), and tumor necrosis factor-alpha (TNFα) (31, 32). KLK5, activated through cleavage by matrix metalloproteinases (MMPs), proteolytically processes antimicrobial peptide precursors into bioactive fragments including LL-37 (33). In patients with rosacea, the aberrant expression and processing of LL37 are considered to be closely associated with the pathogenesis and progression of the disease (34). LL-37 drives rosacea-associated inflammation via multiple mechanisms: recruiting innate immune cells; activating the epidermal growth factor receptor (EGFR) pathway; enhancing neutrophil-derived ROS production; promoting vasodilation and angiogenesis; modulating MCs activity; and increasing cutaneous sensitivity to ultraviolet (UV) radiation (34, 35). Specifically, LL-37 exerts its effects through three downstream signaling pathways, including the JAK/STAT pathway, mechanistic target of rapamycin complex1 (mTORC1), and the NLRP3 inflammasome. The mTORC1 signaling is hyperactivated in rosacea and regulates cathelicidin via a positive feedback loop, in which LL37 stimulates mTORC1 signaling through binding to TLR2, thereby in turn promotes cathelicidin expression itself in human keratinocytes. Furthermore, excessive LL-37 through mTORC1-dependent pathways induces NF-κB activation and promotes the production of rosacea-characteristic inflammatory mediators (including cytokines and chemokines) (36, 37). Animal studies confirm that LL-37 administration induces rosacea-like lesions in mice, with chronic exposure leading to irreversible pathology (32, 35, 38). Additionally, bioinformatic analyses identified the TLR signaling pathway as the most significantly enriched pathway in rosacea. Protein-protein interaction (PPI) network analysis pinpointed TLR2 as a central hub within this pathway (39). These findings indicate that the TLR and cathelicidin pathway play a critical role in the inflammatory mechanisms of rosacea.

Inflammasomes are multiprotein complexes of the innate immune system that trigger inflammatory responses through caspase-1 activation. The NOD-like receptor family, pyrin domain containing 3 (NLRP3), a pattern recognition receptor (PRR) expressed in the cytoplasm of immune cells and keratinocytes, activates immune responses upon cellular damage (40). NLRP3 participates in inflammasome assembly, leading to caspase-1 activation which proteolytically processes pro-inflammatory cytokines into active IL-1β. In rosacea lesions, elevated levels of NLRP3, caspase-1, and IL-1β are observed. Pharmacological inhibition of NLRP3 weaken s LL-37-induced rosacea manifestations, confirming NLRP3 pathogenic role (41–43). Notably, a regulatory crosstalk exists between LL-37 and the inflammasome pathway: TLR2 signaling upregulates pro-IL-1β expression, while LL-37 enhances inflammasome-mediated processing of pro-IL-1β (34).

#### 4.1.2 Innate immune cells

**Figure 2:**
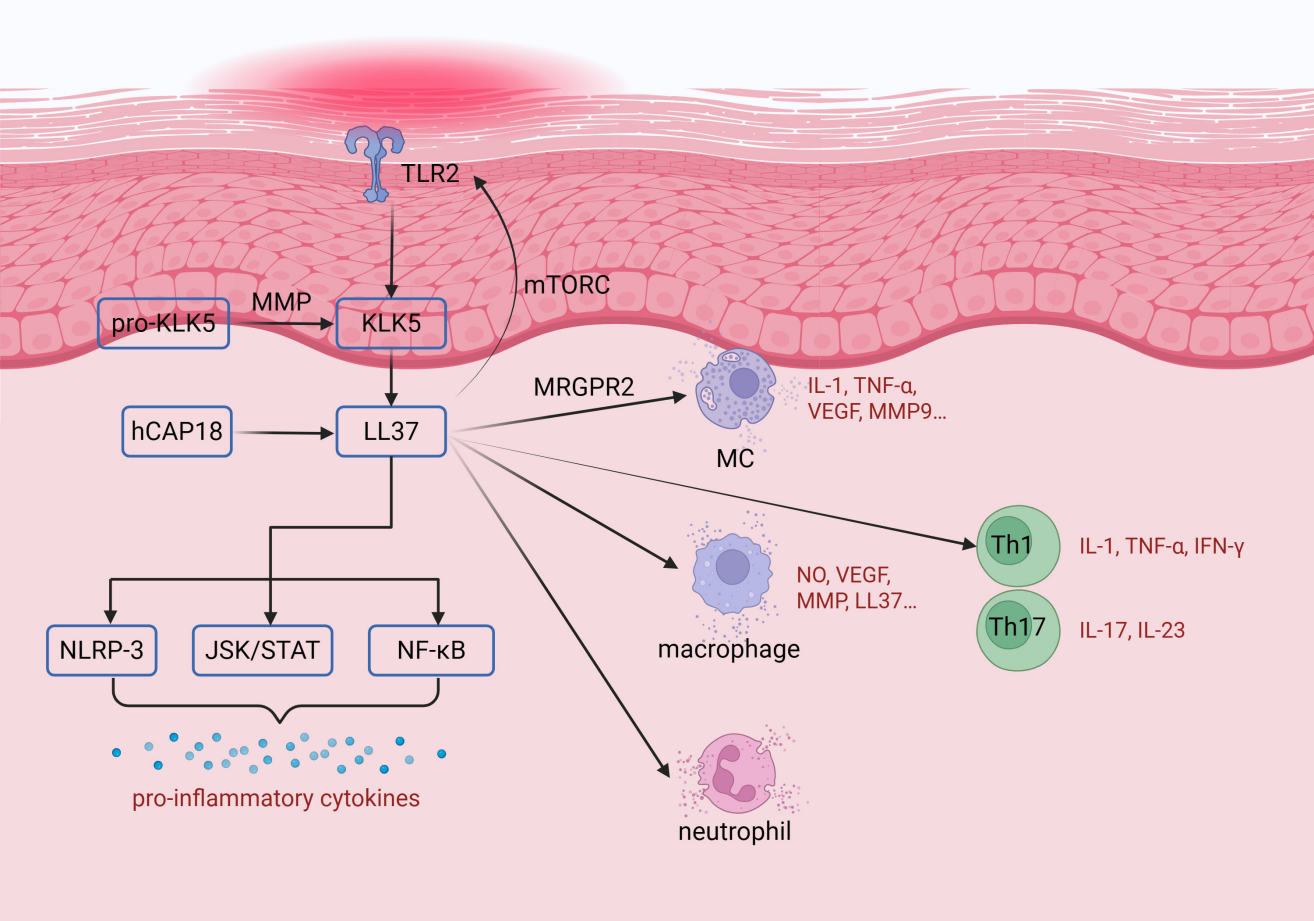
The role of immune dysregulation in the pathogenesis of rosacea. When stimulating factors such as microorganisms, ultraviolet radiation, and psychological stress activate toll-like receptor 2 (TLR2) on keratinocytes, they lead to the expression of kallikrein 5 (KLK5) in rosacea. KLK5, activated through cleavage by matrix metalloproteinases (MMPs), proteolytically processes antimicrobial peptide precursors into LL-37. LL-37 exerts its effects primarily through three downstream signaling pathways, including the Janus protein tyrosine kinase/Signal Transducers and Activators of Transcription (JAK/STAT) pathway, the nuclear factor kappa-light-chain-enhancer of activated B cells (NF-κB) pathway, and the NLRP3 inflammasome pathway. LL-37 can activate various cell types, such as macrophages, neutrophils, T cells, and MCs. Activation of these cells leads to the production of pro-inflammatory cytokines and inflammatory mediators, thereby promoting inflammatory responses, vasodilation, angiogenesis, oxidative stress, and fibrosis. TLR2, toll-like receptor 2; KLK-5, kallikrein 5; hCAP, human cathelicidin; mTOR, mammalian target of rapamycin; IL-, interleukin; TNF-α, tumor necrosis factor; INF, interferon; VEGF, vascular endothelial growth factor; MRGPRX2, Mas-related G-protein coupled receptor member X2; Th-, T helper cell; NLRP3, NLR family pyrin domain containing 3; JAK/STAT, Janus protein tyrosine kinase/Signal Transducers and Activators of Transcription; NF-κB, nuclear factor kappa-light-chain-enhancer of activated B cells; MC, mast cell; MMP, matrix metalloproteinase; NO, Nitric oxide. The figure was created with BioRender.com.

Dysregulation of innate immune cells is observed in the lesional skin of rosacea (**Figure 2**). The number of various innate immune cells—including MCs, macrophages, and neutrophils—is significantly increased (44–46). MCs originate from the bone marrow and, upon maturation, are predominantly distributed in perivascular locations surrounding nerves within most tissues and organs (44, 47). MCs can enhance host defense and mediate local inflammatory damage by rapid degranulation and neutrophil recruitment. The key regulatory target of this biological process is the Mas-related G-protein coupled receptor member X2 (MRGPRX2). This receptor can be specifically activated by the LL-37 and certain drugs, serving as a key molecular hub linking neuropeptide signaling, innate immune responses, and rosacea-associated inflammation. The signaling pathway mediated by MRGPRX2 profoundly reveals the central role of neuro-immune crosstalk in the pathogenesis of rosacea (48, 49). LL-37 and MCs can influence each other. On the one hand, LL-37 not only induces MCs degranulation but also upregulates TRPV4 expression on them, thereby enhancing their capacity to detect PAMPs. On the other hand, activated MCs release matrix metalloproteinase-9 (MMP-9), which further promotes LL-37 generation (22, 44, 50, 51). *In vivo* studies using mast cell-deficient mice have demonstrated that LL-37-induced rosacea-like skin lesions fail to develop, establishing MCs as essential factors in LL-37-mediated rosacea pathogenesis. Recent research has revealed significantly increased MCs density in granulomatous rosacea compared to both erythematotelangiectatic rosacea and healthy control groups. Investigators speculate that this MCs accumulation may represent a chronic manifestation in the later stages of granulomatous rosacea (52). Therefore, MCs play a crucial role in cathelicidin-induced skin inflammation by mediating the secretion of cytokines and bioactive mediators upon stimulation.

### 4.2 Adaptive immune dysregulation

The adaptive immune system is also involved in the occurrence and development of rosacea (**Figure 2**), but our current understanding of its mechanism is still limited. In lesions, CD4+ T cell infiltration levels are elevated, particularly Th1 and Th17 subtypes, Th1 cells secrete elevated levels of IFN-γ and TNF-α, while Th17 cells produce increased amounts of IL-17A, IL-22, IL-6, IL-20, and CCL-20. T cell-derived cytokines such as IL-17A and IL-22 can further promote the recruitment and infiltration of neutrophils. In addition, Th17 cells can induce the high expression of VEGF, thereby facilitating vascular proliferation and dilation; IFN-γ can drive the proliferation and activation of macrophages (46, 53–55). Some chemokines were also upregulated in patients with rosacea, including CXCL8, CXCL1, CXCL2, CXCL5, and CXCL6. These chemokines exhibit angiogenic properties and are able to attract neutrophils and Th17 cells in rosacea (46). Brown, Theodore T et al. compared the T-cell subsets, and plasmacytoid dendritic cells in rosacea and lupus erythematosus. They found that the mean percentage of CD4+ and CD25+ Tregs were higher in rosacea compared with lupus erythematosus (56). This phenomenon may be attributable to either functional impairment of Tregs or a failed compensatory anti-inflammatory mechanism in rosacea patients, thereby contributing to the chronic persistence of the disease. The highly expressed aquaporin 3 (AQP3) in CD4+ T cells is involved in the progression of inflammation. AQP3 is a channel protein that mediates water/glycerol transport. It is crucial for the activation of NF-κB signaling in keratinocytes and can promote the differentiation of T cells into Th17 cells. Finally contributed to the inflammation in rosacea (57). The role of B cells in rosacea has not yet been fully elucidated. The inflammatory infiltrate consists predominantly of lymphocytes, with CD20^+^ B cells comprising 10% to 20% of the cellular population (58). Antibodies can also been observed in rosacea (59). We hypothesize that B cells may participate in the pathogenesis of rosacea through antigen-presenting function to regulate the differentiation and activation of Th1 and Th17 cells, or by abnormally producing autoantibodies which sustain the chronic inflammatory state of rosacea via immune complex deposition or complement system activation. Therefore, the functions of B cell-mediated responses in rosacea warrant further research. Over all, the role of innate immunity in rosacea pathogenesis is well characterized, but the understanding of adaptive immune involvement remains limited. Future research should focus on elucidating the interaction between neural, vascular, microbial, and immune components to further clarity immune dysregulation in rosacea.

## 5 Skin barrier dysfunction

Skin barrier damage is an obvious clinical feature of patients with rosacea. Compared with normal people, patients’ skin shows changes such as increased sensitivity, decreased water content in the stratum corneum, increased transepidermal water loss, and elevated pH (60, 61). Damage of the skin barrier facilitates bacterial colonization on the skin. After bacterial colonization, the combination with antimicrobial peptides may induce and activate rosacea. Medgyesi et al. performed RNA sequencing on the lesions of papulopustular rosacea. The results showed that compared with skin rich in sebaceous glands, the barrier components in rosacea samples changed significantly, including the formation of cornified envelopes and intercellular lipid layers, alterations in desmosomes and tight-junction tissues, as well as changes in barrier antigens and antimicrobial peptides (60). Recently, the overexpression of STAT3 has been considered to be associated with the skin barrier dysfunction in rosacea. Pathological JAK/STAT3 dysregulation subsequently drives pro-inflammatory cytokine production and pathological angiogenesis (62). Changes in cell junctions are more obvious in patients with erythematotelangiectatic and papulopustular rosacea. The mRNA levels of most claudins (CLDN) are decreased, affecting the paracellular pathway between epithelial cells. In *in vitro* experiments, the protein levels of CLDN1, CLDN3, and CLDN5 in keratinocytes are decreased after inducing rosacea (63). A study using scRNA-seq revealed that upregulation of IFNγ signaling may contribute to skin barrier impairment in rosacea, particularly through downregulation of CLDNs in keratinocytes (64). In conclusion, the dysfunction of the skin barrier can lead to changes in the composition of the microbiota, promote immune and inflammatory responses, and play a crucial role in the complex network of the pathogenesis of rosacea.

## 6 Microbial dysbiosis

Skin and gut microbiota play a crucial role in regulating inflammatory responses and immune functions. The composition of microbiota in rosacea patients differs from that in healthy individuals, and this kind of microbial dysbiosis can mediate disease onset by disrupting the skin barrier, triggering inflammatory responses, and secreting bioactive factors.

### 6.1 Skin microbiota

The skin microbiota can prevent the colonization of pathogens, but in some cases, even beneficial or harmless bacteria may become pathogenic. Demodex folliculorum is the main microorganism in the skin, which do not typically cause dermatological problems unless they reach a high load and/or penetrate the dermis (65). Demodex is usually associated with the development of rosacea. It has been observed that Demodex folliculorum tends to accumulate in the central facial area severely affected by rosacea. Moreover, the increase in Demodex density coincides with the exacerbation of rosacea symptoms. This phenomenon indicates that Demodex plays a role in the pathogenesis of rosacea (66, 67). A meta-analysis showed that the colonization rate of Demodex mites in patients with rosacea was as high as 70.4%, while it was only 31.8% in the healthy control group. Both erythematotelangiectatic and papulopustular subtypes of rosacea show elevated Demodex levels (65). Some studies suggest that Demodex may play a role in early inflammation by stimulating TLR - 2 to increase the expression of inflammatory factors (67). In addition to Demodex folliculorum, recent studies have also found that the numbers of Staphylococcus aureus and Streptococcus flora on the faces of rosacea patients have increased (68). Rainer et al. found that Corynebacterium kroppenstedtii was one of the most abundant bacteria in rosacea subjects aged 40-49, while it was absent in the control group (69). The role of Propionibacterium acnes in rosacea is controversial. Some studies suggest that this bacterium has a protective effect on rosacea. Propionibacterium acnes can decompose sebum into free fatty acids, exerting a protective effect on healthy skin and inhibiting the proliferation of pathogenic microorganisms. Rainer et al. reported that Propionibacterium acnes were the most representative bacterium in both patients and the control group, and its number decreased in male patients (70). The controversial role of Propionibacterium acnes may be associated with differences in the cutaneous microenvironment among distinct study populations. It should be noted that the skin microbiome changes with age, gender, environment, and the use of cosmetics and antibiotics, while rosacea severity increases with age—a trend that is related to the changes in the composition and quantity of epidermal microorganisms. (70, 71).

### 6.2 Gut microbiota

In recent years, the association between gut microbiota and rosacea has gradually increased. Helicobacter pylori (HP), a microaerophilic Gram-negative bacterium, colonizes the stomach and duodenum. HP infection has been considered a risk factor for rosacea, but the association between them is weak. Some studies speculate that HP can cause skin inflammation and flushing through cytotoxins and gastrin (72). The concept of the gut-skin axis may explain the pathogenesis of chronic inflammatory skin diseases. This concept posits that skin homeostasis is influenced by the complex interactions among the immune system, metabolic system, and nervous system (73, 74). Based on the findings of Mendelian randomization study, there is a significant causal relationship between gut microbiota and rosacea. Specifically, 16 gut microbial taxa were identified to have either protective or risk effects on rosacea development. Notably, probiotic genera such as Lactobacilli and Bifidobacteria showed protective associations. The causal relationship was found to be unidirectional, indicating that gut microbiota influences rosacea (75). Another Mendelian randomization analysis found that patients with moderate to severe rosacea had a higher Helicobacter pylori infection rate, suggesting that Helicobacter pylori may be a risk factor for rosacea. Meanwhile, the researchers identified that the genus Dialister could reduce the risk of rosacea (76).

## 7 Conclusion

Rosacea is a complex chronic inflammatory skin disease driven by the interaction of genetics, neurovascular function, immunity, skin barrier and microorganisms, with each factor amplifying the others to maintain the disease. In recent years, with the advancement of basic scientific research and the application of emerging technologies, the pathological mechanism of rosacea has gradually been revealed. Advances in genomics, immunology, and microbiology have helped to uncover more information about the susceptibility and inducing factors of the disease. However, the specific pathophysiological mechanism of rosacea has not been fully elucidated. Future research should further explore its molecular and cellular mechanisms, which will provide a theoretical basis for the establishment of personalized treatment strategies and the development of new treatment methods, thereby improving the clinical prognosis of patients.
